# Comorbidity Among Adults With Epilepsy — United States, 2021–2022

**DOI:** 10.5888/pcd21.240313

**Published:** 2024-12-19

**Authors:** Ying Zhou, Rosemarie Kobau, Daniel M. Pastula, Kurt J. Greenlund

**Affiliations:** 1 Epidemiology and Surveillance Branch, Division of Population Health, National Center for Chronic Disease Prevention and Health Promotion, Centers for Disease Control and Prevention, Atlanta, Georgia; 2 University of Colorado School of Medicine and Colorado School of Public Health, Aurora, Colorado

## Abstract

While it is known that epilepsy often co-occurs with psychiatric disorders, few studies have examined nonpsychiatric comorbidity. We analyzed 2021 and 2022 National Health Interview Survey Sample Adult data. Compared with adults with no epilepsy, the 1.2% of US adults (about 3.0 million) with active epilepsy had a higher prevalence of nearly all 21 conditions examined and were more likely to have 4 or more co-occurring chronic conditions. Health care and social service providers can promote healthy behaviors and preventive screening for common comorbidities, improve access to care, and refer people with epilepsy to evidence-based self-management programs.

**Summary**

**What is already known about this topic?**

Epilepsy often co-occurs with psychiatric disorders. Few studies have examined nonpsychiatric comorbidity.

**What is added by this report?**

Among the 1.2% of US adults (about 3.0 million) with active epilepsy, the most prevalent co-occurring conditions were difficulty remembering or concentrating, chronic pain, obesity, and hypertension. Compared with adults with no epilepsy, adults with active epilepsy had a higher prevalence of nearly all chronic conditions examined and were more likely to report 4 or more co-occurring conditions.

**What are the implications for public health practice?**

Primary care, epilepsy health, and social service providers can promote healthy behaviors and preventive screening for common comorbidities, improve access to care, and refer people with epilepsy to evidence-based self-management programs.

## Objective

Epilepsy is a neurologic disorder characterized by recurrent, unprovoked seizures. Although epilepsy often co-occurs with psychiatric disorders (eg, depression) (1,2), few studies have examined nonpsychiatric comorbidity (2,3). A previous study found that a higher percentage of US adults with epilepsy (ie, self-reported doctor-diagnosed epilepsy) reported cardiovascular and respiratory conditions, some inflammatory conditions, and various types of pain than those without epilepsy (4). Beyond managing their epilepsy and its consequences (unemployment, social isolation, limitations in educational attainment), people with epilepsy may face additional challenges associated with comorbidity that can adversely affect their health and quality of life (2,3,5). We provide the most recent US national estimates of nonpsychiatric conditions among adults with epilepsy and those without epilepsy. Our goal is to inform interventions at both the population and individual levels, reduce chronic disease burden, and advance health equity for people with epilepsy.

## Methods

The National Health Interview Survey (NHIS) is an annual cross-sectional household survey of the US civilian, noninstitutionalized population conducted by the National Center for Health Statistics (NCHS) (6). We analyzed 2021 and 2022 Sample Adult data for adults aged 18 years or older who completed the epilepsy section of the questionnaire (N = 57,088) (6).

We applied a validated case-definition of epilepsy status (4,7). Adults who responded “yes” to ever having been told by a doctor or other health professional that they had a seizure disorder or epilepsy were considered as having “any epilepsy.” Adults with any epilepsy and who were currently taking medication to control it, or had 1 or more seizures in the past year, or both were classified as having active epilepsy (4). Those with any epilepsy and who were neither taking medication nor had a seizure in the past year were classified as having “inactive epilepsy.” Remaining respondents were classified as having no epilepsy.

Selected nonpsychiatric conditions in the analysis were those known to be associated with epilepsy and of interest to epilepsy providers (Table 1) (2–4). We included 15 chronic conditions available in both 2021 and 2022 NHIS data sets and 5 chronic conditions available only in 2021. In addition to dementia, we included a related symptom, difficulty remembering or concentrating, as an available study variable given its saliency as a potential medication side effect and to identify potential early symptoms of undiagnosed dementia. Given the ongoing COVID-19 pandemic during the study period, we also examined the prevalence of doctor-diagnosed COVID-19 by epilepsy status in both years as well as whether the respondent ever had a positive COVID-19 test in 2022. This would provide insight into whether people with different epilepsy statuses were affected similarly by an infectious disease and by the selected chronic conditions. For conditions available only in 2021 or 2022, we used the original survey weight. For conditions available in both years, we combined data from each year and created a new weight variable by dividing the original weight by 2. For chronic conditions available in both 2021 and 2022, we calculated percentages of adults with 0, 1, 2, 3, or 4 or more comorbid conditions by epilepsy status.

**Table 1:** Questions for Comorbid Conditions — National Health Interview Survey, United States, 2021 and 2022

Condition	Question text	Note
Hypertension	Have you EVER been told by a doctor or other health professional that you had hypertension, also called high blood pressure?	If the answer is yes, then the respondent is considered to have this condition.
Coronary heart disease	1. Have you EVER been told by a doctor or other health professional that you had coronary heart disease? 2. Have you EVER been told by a doctor or other health professional that you had angina, also called angina pectoris? 3. Have you EVER been told by a doctor or other health professional that you had a heart attack, also called myocardial infarction?	If the answer to any of the 3 questions is yes, the respondent is considered to have coronary heart disease.
Stroke	Have you EVER been told by a doctor or other health professional that you had a stroke?	If the answer is yes, then the respondent is considered to have this condition.
High cholesterol	Have you EVER been told by a doctor or other health professional that you had high cholesterol?	If the answer is yes, then the respondent is considered to have this condition.
Diabetes	Not including gestational diabetes and prediabetes, has a doctor or other health professional EVER told you that you had diabetes?	If the answer to this question is yes, then the respondent is considered to have this condition.
Prediabetes	Has a doctor or other health professional EVER told you that you had prediabetes or borderline diabetes?	If the answer is yes, then the respondent is considered to have this condition.
Overweight	BMI = weight in kg/height in m^2^. For both men and women, overweight is BMI ≥25 to <30; obesity is BMI ≥30.	Based on self-reported height and weight.
Obesity		
Cancer	Have you EVER been told by a doctor or other health professional that you had cancer or a malignancy of any kind?	If the answer is yes, then the respondent is considered to have this condition.
Asthma	Have you EVER been told by a doctor or other health professional that you had asthma?	If the answer is yes, then the respondent is considered to have this condition.
Chronic obstructive pulmonary disease	Have you EVER been told by a doctor or other health professional that you had chronic obstructive pulmonary disease, COPD, emphysema, or chronic bronchitis?	If the answer is yes, then the respondent is considered to have this condition.
Arthritis	Have you EVER been told by a doctor or other health professional that you had some form of arthritis, rheumatoid arthritis, gout, lupus, or fibromyalgia?	If the answer is yes, then the respondent is considered to have this condition.
Difficulty remembering or concentrating	Do you have difficulty remembering or concentrating?	If the answer is yes, then the respondent is considered to have this condition.
Dementia	Have you EVER been told by a doctor or other health professional that you had dementia, including Alzheimer's disease?	If the answer is yes, then the respondent is considered to have this condition.
Immunosuppression	1. In the past 12 months, have you taken prescription medication or had any medical treatments that a doctor or other health professional told you would weaken your immune system? 2. Do you currently have a health condition that a doctor or other health professional told you weakens the immune system, even without related medications or treatments?	Respondent is considered to have this condition if the answer is “Yes” to at least 1 of these 2 questions.
Chronic pain on most days or every day	In the past 3 months, how often did you have pain? Would you say never, some days, most days, or every day?	Respondent is considered to have this condition if the answer is “Most days” or “Every day.” Available only in 2021 data.
Migraine	Over the past 3 months, how much have you been bothered by headache or migraine?	This question is only for the subset of respondents who did not answer “Never” to the above question on chronic pain. Respondent is considered to have this condition if the answer is “A little”, “A lot” or “Somewhere in between a little and a lot.” Available only in 2021 data.
Weak/failing kidney	Have you EVER been told by a doctor or other health professional that you had weak or failing kidneys?	If the answer is yes, then the respondent is considered to have this condition. Available only in 2021 data.
Liver condition	Have you EVER been told by a doctor or other health professional that you had cirrhosis or any other kind of long-term liver condition?	If the answer is yes, then the respondent is considered to have this condition. Available only in 2021 data.
Current skin allergy	The next question is about an allergic skin condition. Do you get an itchy rash due to eczema or atopic dermatitis?	If the answer is yes, then the respondent is considered to have this condition. Available only in 2021 data.
COVID-19 (doctor diagnosed)	Has a doctor or other health professional ever told you that you had or likely had coronavirus or COVID-19?	If the answer is yes, then the respondent is considered to have this condition.
COVID-19 (positive test ever)	Did you ever take a test that showed you had coronavirus or COVID-19? Testing includes antibody or blood tests as well as other forms of testing for COVID-19, such as a nasal swabbing or throat swabbing.	If the answer is yes, then the respondent is considered to have this condition. Available only in 2022 data.

Abbreviation: BMI, body mass index.

We used R version 4.3.0 (R Foundation) and its survey package (version 4.1) to conduct analyses accounting for the complex survey data. We estimated prevalence and 95% CIs for epilepsy status by using the 2000 US Census Bureau projected US adult population (2000 standard population) with 3 age groups (18‒44 y, 45‒64 y, ≥65 y), consistent with the NCHS guidance on age standardization (8,9). We evaluated the statistical significance of percentage differences by epilepsy status with the χ^2 ^test at the .05 level. We examined and compared both crude prevalence estimates and age-standardized estimates. Results focused on age-standardized estimates. Estimates were suppressed if they did not meet NCHS data presentation standards for proportions (10).

## Results

In 2021 and 2022, 1.8% of US adults (population estimate of 4.6 million) were classified as having any epilepsy, including 1.2% (3.0 million) with active epilepsy (Table 2). Adults with active (43.6%) or inactive (40.9%) epilepsy were more likely to report 4 or more chronic conditions than adults without epilepsy (21.2%) (Figure).

**Table 2:** Age-Standardized Prevalence^a^ of Selected Nonpsychiatric Conditions Among Adults, by Epilepsy Status, National Health Interview Survey, United States, 2021 and 2022^b^

Condition^c^	Any epilepsy		Active epilepsy			Inactive epilepsy			No epilepsy	
	No.^d^	%^a^ (95% CI)	No.	% (95% CI)	*P *value^e^	No.	% (95% CI)	*P *value^e^	No.	% (95% CI)
Total^f^	1,006	1.8 (1.7‒2.0)	635	1.16 (1.0‒1.3)	Not applicable	364	0.66 (0.6‒0.7)	Not applicable	55,935	98.2 (98.0‒98.3)
Hypertension	461	38.9 (35.2‒42.8)	292	38.1 (33.7‒42.7)	<.001	164	39.7 (33.4‒46.2)	<.001	20,291	28.4 (27.9‒28.8)
Coronary heart disease	147	10.6 (8.7‒12.9)	87	10.2 (7.7‒13.2)	<.001	58	11.3 (7.8‒15.6)	.001	4,441	5.5 (5.3‒5.7)
Stroke	158	13.3 (11.1‒15.8)	114	15.7 (12.5‒19.3)	<.001	41	8.5 (5.3‒12.7)	<.001	1,857	2.2 (2.1‒2.3)
High cholesterol	382	31.1 (27.7‒34.8)	240	30.5 (26.3‒35.0)	.003	139	32.2 (26.3‒38.4)	.006	17,390	24.1 (23.7‒24.5)
Diabetes	153	12.5 (10.2‒15.1)	101	12.4 (9.8‒15.5)	.004	49	11.7 (7.6‒16.9)	.12	5,913	8.4 (8.1‒8.7)
Prediabetes	220	19.5 (16.5‒22.8)	132	17.9 (14.5‒21.8)	.009	82	21.1 (16.0‒.26.9)	.003	8,965	13.2 (12.9‒13.6)
Overweight	303	28.6 (25.1‒32.4)	187	28.2 (23.8‒32.8)	.04	115	29.7 (24.3‒35.7)	.27	18,849	32.9 (32.3‒33.4)
Obesity	385	40.7 (36.9‒44.6)	232	38.6 (34.0‒43.5)	.006	147	43.5 (37.2‒49.8)	<.001	17,624	32.1 (31.4‒32.7)
Cancer	165	12.7 (10.4‒15.3)	104	13.5 (10.6‒16.9)	<.001	60	11.4 (7.9‒15.7)	.07	6,901	8.2 (8.0‒8.4)
Asthma	244	23.8 (20.6‒27.3)	152	23.5 (19.6‒27.8)	<.001	89	24.1 (18.7‒30.2)	<.001	7,684	14.2 (13.8‒14.6)
COPD	153	12.5 (10.3‒14.9)	97	13.4 (10.4‒16.8)	<.001	54	10.3 (7.0‒14.6)	<.001	3,040	3.9 (3.7‒4.1)
Arthritis	421	35.2 (31.6‒38.9)	276	36.1 (31.7‒40.7)	<.001	142	33.3 (27.2‒39.8)	<.001	14,449	18.5 (18.1‒18.9)
Difficulty remembering	507	50.5 (46.7‒54.3)	350	55.8 (50.8‒60.7)	<.001	151	40.6 (34.3‒47.2)	<.001	11,770	19.1 (18.6‒19.6)
Dementia	41	3.5 (2.1‒5.3)	33	4.5 (2.5‒7.4)	<.001	7	—	—	681	0.8 (0.7‒0.9)
Immunosuppression	172	16.5 (13.9‒19.4)	107	16.8 (13.5‒20.6)	<.001	62	15.4 (11.1‒20.7)	<.001	4,133	6.5 (6.3‒6.8)
Chronic pain on most days or every day	222	41.3 (36.0‒46.7)	133	40.2 (33.8‒46.8)	<.001	87	42.3 (33.5‒51.4)	<.001	6,436	20.0 (19.4‒20.6)
Migraine^g^	201	55.4 (49.2‒61.4)	127	59.7 (51.7‒67.2)	<.001	72	47.4 (37.4‒57.5)	.48	7,000	43.9 (43.0‒44.9)
Weak/failing kidney	38	6.8 (4.1‒10.6)	21	—	—	16	—	—	960	2.8 (2.6‒3.0)
Liver condition	16	—	10	—	—	5	—	—	269	0.8 (0.7‒1.0)
Current skin allergy	77	14.8 (11.3‒18.9)	46	13.3 (9.3‒18.3)	.33	30	17.1 (10.0‒26.4)	.08	3,153	10.7 (10.2‒11.1)

Abbreviations: COPD, chronic obstructive pulmonary disease; —, estimates suppressed because they did not meet NCHS data presentation standards for proportions (10).

a Age-standardized prevalence was calculated by using the 2000 US Census Bureau–projected US adult population with 3 age groups (18‒44 y, 45‒64 y, ≥65 y) (https://www.cdc.gov/nchs/data/statnt/statnt20.pdf).

b Sample size and response rates for NHIS, 2021 (n = 29,482; response rate = 50.9%) and 2022 (n = 27,651; response rate = 47.7%). In 2021 and 2022, 45 respondents did not answer supplementary survey question on epilepsy and were not included in this analysis. Among respondents with any epilepsy, 7 had missing information and could not be categorized as active or inactive epilepsy. Respondents with missing complex survey design parameters or who did not report age were not included in the table.

c Questions for assessing comorbid conditions are in Table 1.

d Unweighted.

e *P* value is from the χ^2^ test using adults with no epilepsy as reference. When the χ^2^ test was performed comparing adults with any epilepsy, with no epilepsy as reference, all *P* values were <.001, except for overweight (*P* = .02) and current skin allergy (*P* = .05).

f The estimate of the total US adult population with any epilepsy is 4.6 million: 2.95 million with active epilepsy and 1.66 million with inactive epilepsy.

g Percentage with migraine is only among respondents who had chronic pain, that is, respondents who answered “Some days,” “Most days,” or “Every day” to the question “In the past 3 months, how often did you have pain?”

**Figure:**
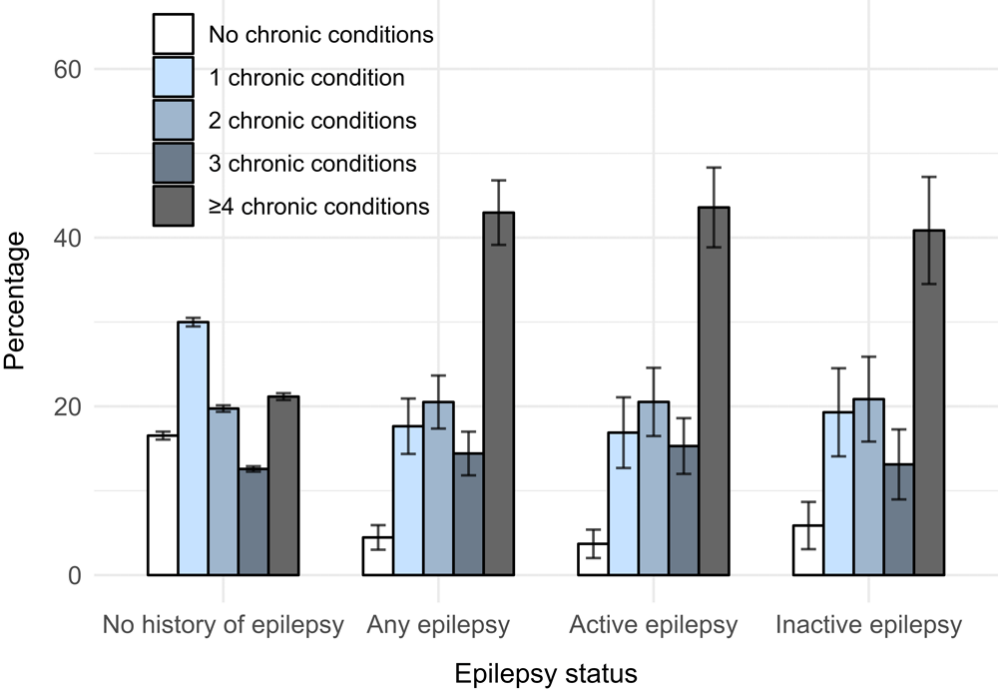
Percentage of adults with nonpsychiatric chronic conditions, by number of conditions and epilepsy status, National Health Interview Survey, United States, 2021 and 2022. Error bars indicate 95% CIs. Percentage was age standardized by using the 2000 US Census Bureau–projected US adult population with 3 age groups (18‒44 y, 45‒64 y, ≥65 y) (8). Chronic conditions included were hypertension, coronary heart disease, stroke, high cholesterol, diabetes, prediabetes, overweight, obesity, cancer, asthma, chronic obstructive pulmonary disease, arthritis, difficulty remembering or concentrating, dementia, and immunosuppression. Adults with epilepsy (active or inactive) were more likely to report 4 or more chronic conditions than adults without epilepsy.

**Table:** 

Epilepsy status	Number of chronic conditions	% (95% CI)
No history of epilepsy	No chronic conditions	16.5 (16.1-17.0)
No history of epilepsy	1 chronic condition	30.0 (29.5-30.5)
No history of epilepsy	2 chronic conditions	19.7 (19.4-20.1)
No history of epilepsy	3 chronic conditions	12.6 (12.3-12.9)
No history of epilepsy	≥4 chronic conditions	21.2 (20.8-21.6)
Any epilepsy	No chronic conditions	4.5 (3.0-5.9)
Any epilepsy	1 chronic condition	17.6 (14.4-20.9)
Any epilepsy	2 chronic conditions	20.5 (17.4-23.7)
Any epilepsy	3 chronic conditions	14.4 (11.8-17.0)
Any epilepsy	≥4 chronic conditions	43.0 (39.1-46.8)
Active epilepsy	No chronic conditions	3.7 (2.0-5.4)
Active epilepsy	1 chronic condition	16.9 (12.7-21.1)
Active epilepsy	2 chronic conditions	20.5 (16.5-24.6)
Active epilepsy	3 chronic conditions	15.3 (12.0-18.6)
Active epilepsy	≥4 chronic conditions	43.6 (38.9-48.3)
Inactive epilepsy	No chronic conditions	5.9 (3.1-8.7)
Inactive epilepsy	1 chronic condition	19.3 (14.1-24.5)
Inactive epilepsy	2 chronic conditions	20.9 (15.8-25.9)
Inactive epilepsy	3 chronic conditions	13.1 (9.0-17.3)
Inactive epilepsy	≥4 chronic conditions	40.9 (34.5-47.2)

Among adults with active epilepsy, the most prevalent comorbidities were difficulty remembering (55.8%), chronic pain (40.2%), obesity (38.6%), and hypertension (38.1%) (Table 2). Compared with adults with no epilepsy, adults with active epilepsy had a significantly higher prevalence of all selected chronic conditions except for overweight and current skin allergy. The largest absolute differences between those with active versus no epilepsy were difficulty remembering (55.8% vs 19.1%), chronic pain (40.2% vs 20.0%), and arthritis (36.1% vs 18.5%). The largest relative differences between people with active versus no epilepsy were stroke (15.7% vs 2.2%) and chronic obstructive pulmonary disease (13.4% vs 3.9%). Fewer adults with active epilepsy reported no comorbid conditions (3.7%) than those without epilepsy (16.5%). About the same percentages of adults with active epilepsy (22.8%) reported doctor-diagnosed COVID-19 infection as those without epilepsy (21.1%). In 2022, a slightly lower percentage of adults with active epilepsy (36.1%) reported ever having a positive COVID-19 test than adults without epilepsy (39.3%), though the difference was not statistically significant.

Compared with adults with inactive epilepsy, adults with active epilepsy had higher percentages of stroke (15.7% vs 8.5%; *P* = .003), difficulty remembering (55.8% vs 40.6%; *P* < .001), and migraine (59.7% vs 47.4%, *P* = .05). People with inactive epilepsy had similar prevalence as those with active epilepsy for the remaining comorbid conditions. Compared with adults without epilepsy, most conditions were more prevalent among adults with inactive epilepsy except for diabetes, overweight, cancer, migraine, and current skin allergy.

## Discussion

Our study’s findings underscore the high burden of multiple (2 or more) chronic conditions among people with active epilepsy and potential gaps in chronic disease prevention (11). Our findings were similar to the earlier study using 2010 NHIS data, even though some selected chronic conditions differed (4). Our findings also supplement other studies despite differing methodologies (2,3). In contrast with marked differences in chronic comorbidities by epilepsy status, there were no differences in COVID-19 infection by epilepsy status. Previous studies indicate mixed conclusions about the susceptibility of patients with epilepsy for COVID-19 infection, with speculation that epilepsy might not increase risk for COVID-19 infection (12). The high burden of comorbidity among adults with inactive epilepsy is suggestive of the lifelong adverse effect of an epilepsy diagnosis earlier in life (5).

The association of epilepsy with other chronic medical conditions may reflect direct causal relationships (eg, stroke), potential shared underlying etiology (eg, migraine), treatment (eg, medication side effects), lifestyle considerations (eg, concerns about exercise), and the interplay between individual and social risk factors (eg, access to care; family income) (2,13). Many comorbid conditions are preventable through nonpharmacologic interventions (eg, healthy diet, exercise, quality sleep), treatable at the primary care level, or improvable with epilepsy self-management programs (such as HOBSCOTCH for memory [14]).

Our study has several limitations. First, data were based on self-reports and subject to reporting and recall bias. There is also potential misclassification of epilepsy, because certain acute seizures or nonepileptic seizures might have been misclassified as epilepsy. Even so, an earlier study evaluated epilepsy identification based on self-report and found only minimal likelihood of bias and misclassification (7). Second, although social determinants of health, such as education and income, are associated with epilepsy and can affect the prevalence of epilepsy comorbidities (eg, stroke) (15), we did not stratify by social determinants of health because of small sample sizes. Third, given the extensive research already available, we omitted psychiatric comorbidity, which likely underestimated the overall multimorbidity burden. Fourth, we did not account for the frequency of health care visits by people with epilepsy, which might lead to more disease diagnoses, thereby increasing prevalence of comorbidity. Finally, the cross-sectional design of the survey prohibited the examination of temporal associations or causation between epilepsy and the co-occurring disorders.

Primary care, epilepsy health, and social service providers can promote healthy behaviors and preventive screening for common comorbidities, improve access to care, and refer people with epilepsy to evidence-based epilepsy self-management programs shown to improve disease management skills and health outcomes. These efforts may improve the quality of life for people with epilepsy, while simultaneously reducing the health care burden and advancing health equity for people with epilepsy.
